# Supplementary material for: Study on the Adaptive Regulation of Light on the Stress Response of Mandarin Fish (Siniperca chuatsi) with Re-Feeding after Starvation
Source: Animals (Basel). 2023 Aug 13;13(16):2610. doi: 10.3390/ani13162610 (PMC10451258; doi:10.3390/ani13162610)
Supplement: Supplementary file 1 [file animals-13-02610-s001.zip › animals-2524567-supplementary.pdf]

Table S1. Nutritional composition of feed

Item	Crude protein	Crude lipid	Crude fibre	Ash	Water	Phosphorus	Sodium chloride	Calcium	Lysine	Methionine
Content (%)	≥ 45	≥ 4.0	≤ 8.0	≤ 22.0	≤ 10.0	≥ 1.2	≤ 3.0	≥ 2.5	≥ 2.5	≥ 1.2

Table S2. Breeding environment data

Item	Group 1	Group 2	Group 3	Group 4	Group 5
Light intensity (lx)	21.04 ± 2.72^{Aa}	18.44 ± 3.00^{ABb}	15.75 ± 2.88^{BCc}	13.43 ± 2.35^{CDd}	11.15 ± 2.01^{De}
Temperature (℃)	22.31 ± 0.30	22.40 ± 0.41	22.32 ± 0.25	22.38 ± 0.41	22.36 ± 0.50
Dissolved oxygen (mg L^{-1})	8.54 ± 0.02	8.54 ± 0.01	8.58 ± 0.09	8.54 ± 0.02	8.55 ± 0.02
NH_4^+ (mg L^{-1})	0.01 ± 0.00	0.01 ± 0.00	0.01 ± 0.00	0.01 ± 0.00	0.01 ± 0.00
NO_2^- (mg L^{-1})	-	-	-	-	-

Note: Different lowercase letters represent significant differences, while the same uppercase letter represents no significant differences.

Table S3. The stress related indicators and digestive enzymes of *Siniperca chuatsi* before hunger

Tissue	total protein (μg mL^{-1})	ACP (King unit g^{-1} prot)	AKP (King unit g^{-1} prot)	LDH (U g^{-1} prot)	SOD (U mg^{-1} prot)	CAT (U mg^{-1} prot)	GPT (U g^{-1} prot)	GOT (U g^{-1} prot)	Protease (U ml^{-1})	Lipase (U g^{-1} prot)	α-amylase (U mg^{-1} prot)
Gill	4062.42 ± 231.21	5.89 ± 0.42	4.28 ± 0.45	62.78 ± 1.36	26.93 ± 0.29	42.11 ± 1.27	206.43 ± 5.64	266.64 ± 5.42	na	na	na
Brain	5628.37 ± 169.35	7.73 ± 0.31	4.46 ± 0.17	33.02 ± 0.47	25.86 ± 1.01	24.31 ± 1.25	151.28 ± 2.26	298.34 ± 3.72	na	na	na
Intestine	5552.68 ± 331.64	45.85 ±0.66	100.22 ± 0.78	42.85 ± 0.66	20.02 ± 0.34	30.59 ± 0.56	122.56 ± 3.72	264.33 ± 3.17	1.51 ± 0.03	651.41 ± 23.15	0.51 ± 0.09
Stom	4162.51 ±	16.43 ±	3.12 ± 0.24	61.34 ±	24.53 ±	20.64 ±	151.42 ±	297.43 ±	1.64 ± 0.11	1165.33 ±	0.42 ± 0.07

ach	215.21	0.23		1.20	0.67	0.96	3.22	6.38		34.27	
Kidney	9015.46 ± 178.56	10.22 ± 0.11	4.73 ± 0.16	40.36 ± 0.72	16.42 ± 0.86	33.72 ± 1.12	76.85 ± 6.31	142.21 ± 2.65	na	na	na
Liver	9754.81 ± 316.11	12.92 ± 0.35	1.47 ± 0.35	12.03 ± 0.17	16.36 ± 0.11	67.68 ± 2.54	86.54 ± 2.41	134.61 ± 3.97	na	na	na
Plasma	30451.47 ± 541.33	14.37 ± 0.16	0.21 ± 0.06	3.27 ± 0.11	3.56 ± 0.09	6.64 ± 0.25	22.23 ± 1.56	28.19 ± 0.77	na	na	na

Note: “na” represents there was no relevant data. *n* = 5.

Formula for calculating enzyme activity

Total protein contents (ug ml^{-1}) = (OD*a* - OD*b*) / (OD*c* - OD*b*) * A * B

Note: OD*a* represents the absorbance value of the sample. OD*b* represents the absorbance value of the blank group. OD*c* represents the absorbance value of the standard product. A represents the standard product concentration (524 μg/mL). B represents the sample dilution ratio.

Protease (U ml^{-1}) = (OD*a* - OD*b*) / (OD*c* - OD*d*) * A * B / C * (D / E / F)

Note: OD*a* represents the absorbance value of the sample. OD*b* represents the absorbance value of the control group. OD*c* represents the absorbance value of the standard product. OD*d* represents the absorbance value of the blank group. A represents the standard product concentration (50μg/mL). B represents the sample dilution ratio. C represents the protein concentration of the sample (g prot/L). D represents the total volume of the reaction solution (0.64 ml). E represents the sampling quantity (0.04 ml). F represents the reaction time (10 min).

Lipase (U g^{-1} prot) = (OD*a* - OD*b*) / (OD*c* - OD*b*) * A * B / C

Note: OD*a* represents the absorbance change value of the sample. OD*b* represents the absorbance change value of the blank group. OD*c* represents the absorbance change value of the standard product. A represents the standard product activity (45.8 U/L). B represents the sample dilution ratio. C represents the protein concentration of the sample (g prot/L).

α-amylase (U mg^{-1} prot) = (OD*b* - OD*a*) / OD*b* * (0.4 * 0.5 / 10) * (30/7.5) / A * B / C

Note: OD*a* represents the absorbance value of the sample. OD*b* represents the absorbance value of the control group. A represents the sampling quantity (0.1 ml). B represents the sample dilution ratio. C represents the protein concentration of the sample (mg prot/mL).

ACP (King unit g^{-1} prot) = (OD*a* - OD*b*) / (OD*c* - OD*b*) * A * B / C

Note: OD*a* represents the absorbance value of the sample. OD*b* represents the absorbance value of the blank control. OD*c* represents the absorbance value of the standard product. A represents the standard product concentration (0.1 mg/mL). B represents the sample dilution ratio. C represents the protein concentration of the sample (g prot/ml).

AKP (King unit g^{-1} prot) : It's the same as ACP's.

LDH (U g^{-1} prot) = (OD*a* - OD*b*) / (OD*c* - OD*d*) * A * B / C

Note: OD*a* represents the absorbance value of the sample. OD*b* represents the absorbance value of the control group. OD*c* represents the absorbance value of the standard product. OD*d* represents the absorbance value of the blank group. A represents the standard product concentration (0.2μmol/mL). B represents the sample dilution ratio. C represents the protein concentration of the sample (g prot/ml).

SOD (U mg^{-1} prot) = A / 0.5 * B / C *D; A (%) = [(OD*c* - OD*d*) - (OD*a* - OD*b*)]/ (OD*c* - OD*d*) * 100%

Note: A represents the inhibition rate of SOD. B represents the sample dilution ratio. C represents the protein concentration of the sample (mg prot/ml). D represents the dilution ratio of the reaction system, which is the total reaction volume/sampling volume (0.24 / 0.02). OD*a* represents the absorbance value of the sample. OD*b* represents the absorbance value of the blank in the experimental group. OD*c* represents the absorbance value of the control group. OD*d* represents the absorbance value of the blank in control group.

CAT (U mg^{-1} prot) = (OD*b* - OD*a*) * 271 / (60 *A) * B / C

Note: OD*a* represents the absorbance value of the sample. OD*b* represents the absorbance value of the control group. A represents the sampling quantity (ml). B represents the sample dilution ratio. C represents the protein concentration of the sample (mg prot/ml).

GPT ($U\ g^{-1}$ prot) = A * 0.482 * B / C; A ($U\ L^{-1}$) = y = a + b * (OD*a* - OD*b*) + c * (OD*a* - OD*b*) ^ 1.5 + d * (OD*a* - OD*b*) ^ 2.5 + e * (OD*a* - OD*b*) ^ 3

Note: A represents the enzyme activity of the homogenate assay solution. B represents the sample dilution ratio. C represents the protein concentration of the sample (g prot/L). y represents the standard curve, R^2 = 0.99999, and the a, b, c, d, e are 0.01, 170.62, 1206.07, -4614.65 and 6556.84, respectively. OD*a* represents the absorbance value of the sample. OD*b* represents the absorbance value of the control group.

GOT ($U\ g^{-1}$ prot) = A * 0.482 * B / C; A ($U\ L^{-1}$) = y = a + b * (OD*a* - OD*b*) + c * (OD*a* - OD*b*) ^ 2.5 + d * (OD*a* - OD*b*) ^ 3

Note: A represents the enzyme activity of the homogenate assay solution. B represents the sample dilution ratio. C represents the protein concentration of the sample (g prot/L). y represents the standard curve, R^2 = 0.99999, and the a, b, c and d are 0.02, 303.10, -2060.90 and 14023.06, respectively. OD*a* represents the absorbance value of the sample. OD*b* represents the absorbance value of the control group.
